# Shared Decision-Making in the Management of Schizophrenia: A Systematic Review

**DOI:** 10.7759/cureus.87069

**Published:** 2025-06-30

**Authors:** Kierra N Jackson, Alyona G Lee, Jasmin M Ali, Shielene Vargas, Pamela Ulloa-Franco, Travon Manning, Zainab Jimoh, M. Mahbub Hossain

**Affiliations:** 1 College of Medicine, Tilman J. Fertitta Family College of Medicine, Houston, USA

**Keywords:** decision-making process, medication adherence strategies, schizophrenia, schizo-phrenia, schizophrenia spectrum, shared decision-making

## Abstract

Schizophrenia is a chronic mental disorder that affects an individual’s emotional, cognitive, and behavioral well-being. Schizophrenia is characterized by poor treatment adherence, frequent relapses, and worsening symptoms, often leading to increased hospitalizations. Shared decision-making (SDM) has been proposed to enhance adherence by involving patients and their caregivers in collaborative treatment decisions. However, the impact of SDM on adherence, quality of life, and mental health outcomes remains uncertain. This review evaluates the effectiveness of SDM interventions on treatment adherence in individuals with schizophrenia and explores health-related outcomes, including quality of life, mental health, self-efficacy, trust in healthcare providers, and self-regard. Following the Preferred Reporting Items for Systematic Reviews and Meta-Analyses (PRISMA) guidelines, PubMed, CINAHL, and Web of Science were systematically searched. Randomized controlled trials, quasi-experimental, and observational studies assessing adherence were included. Data extraction was performed by two independent reviewers, and study quality was assessed using the Cochrane Risk of Bias Tool. Eight studies involving 2,314 participants met the inclusion criteria. SDM interventions improved treatment adherence in most studies, with rates increasing by 9% (78% vs. 69%, p < 0.01). Quality of life, measured by the World Health Organization Quality-of-Life Scale Abbreviated Version (WHOQOL-BREF), improved by 8.03 points (p < 0.002), and self-esteem, measured via the Rosenberg Scale, increased by 4.06 points (p < 0.001). Additional outcomes included a statistically significant increase in problem-solving ability scores in the SDM group, from 106.68 to 124.00 (n = 29, p < 0.001), while perceived autonomy support, patient activation, and confidence in communicating with providers were also notably higher for SDM participants (p < 0.05). In conclusion, SDM interventions demonstrate significant benefits in improving treatment adherence, quality of life, and various psychological outcomes in individuals with schizophrenia. The findings suggest that SDM fosters greater patient engagement, enhances self-efficacy, and strengthens trust in healthcare providers, ultimately leading to better mental health management. Further research is needed to explore long-term effects and optimize SDM strategies for sustained adherence and improved patient outcomes.

## Introduction and background

Schizophrenia is a chronic and severe mental disorder characterized by profound disruptions in thinking, perception, emotional regulation, and behavior, affecting approximately 0.3-1.6% of the US adult population annually [1,2]. It manifests through positive symptoms such as hallucinations and delusions, negative symptoms including diminished emotional expression and avolition, and cognitive impairments that affect memory, executive function, and processing speed [3,4]. These cognitive deficits significantly impact daily functioning, reducing treatment adherence and overall quality of life [5]. The estimated burden of schizophrenia in the US doubled between 2013 and 2019, reaching $343.2 billion [6]. Commercially insured patients with schizophrenia have inpatient and ER admission rates more than 13 times higher than those without the condition [6]. High healthcare utilization, along with costs from disability-related caregiving and unemployment, highlights the need for better management strategies. To reduce the health and economic burdens of schizophrenia and schizoaffective disorder, more effective treatment and support systems are essential.

Patient-centered care has become increasingly central to schizophrenia management as our understanding of psychiatric disorders continues to evolve [5]. Managing schizophrenia is challenging due to its complex pathophysiology. While dopaminergic pathways play a key role, other undiscovered mechanisms are potentially involved [7]. This complexity highlights the need for effective long-term pharmacological and psychosocial interventions to ensure patient safety [7]. A critical component of this management is treatment adherence, yet nonadherence remains a significant challenge, with rates reaching up to 50% among patients with schizophrenia [5]. Common barriers include medication side effects, lack of insight into the illness, stigma, and poor communication between patients and healthcare providers [5]. Shared decision-making (SDM) has emerged as a potential approach to overcoming these challenges by fostering patient engagement, improving adherence, and enhancing overall treatment outcomes in schizophrenia care [8].

SDM is a collaborative approach wherein clinicians and patients work together to make informed healthcare decisions based on the best available evidence and patient preferences [8]. While SDM is well-established in general medicine, its integration into psychiatric care - particularly schizophrenia management - remains limited [9]. Key barriers include cognitive impairments that affect decision-making capacity, healthcare providers’ skepticism about patient competence, and structural challenges within mental health services [9]. Despite these obstacles, emerging SDM interventions - such as structured decision aids, psychoeducation, and digital health tools - have demonstrated potential in enhancing patient autonomy and treatment adherence [10-12]. By fostering patient engagement, SDM addresses critical challenges such as nonadherence and communication gaps while upholding ethical principles of autonomy and patient-centered care [10-12]. Research further suggests that these interventions not only improve patient satisfaction but, more importantly, have the potential to enhance long-term clinical outcomes [10-12].

This systematic review examines whether integrating SDM into schizophrenia care can enhance both short- and long-term patient adherence, informing future clinical practice and policy. We critically evaluate current research, identify gaps in the literature, and provide insights to guide clinical and policy development. By assessing the benefits and limitations of SDM in schizophrenia management, this review aims to contribute to more effective and holistic psychiatric treatment approaches. Given recent literature on SDM interventions and their impact on schizophrenia care, we hypothesize that structured SDM approaches may improve adherence and clinical outcomes.

Methods

**Figure 1:**
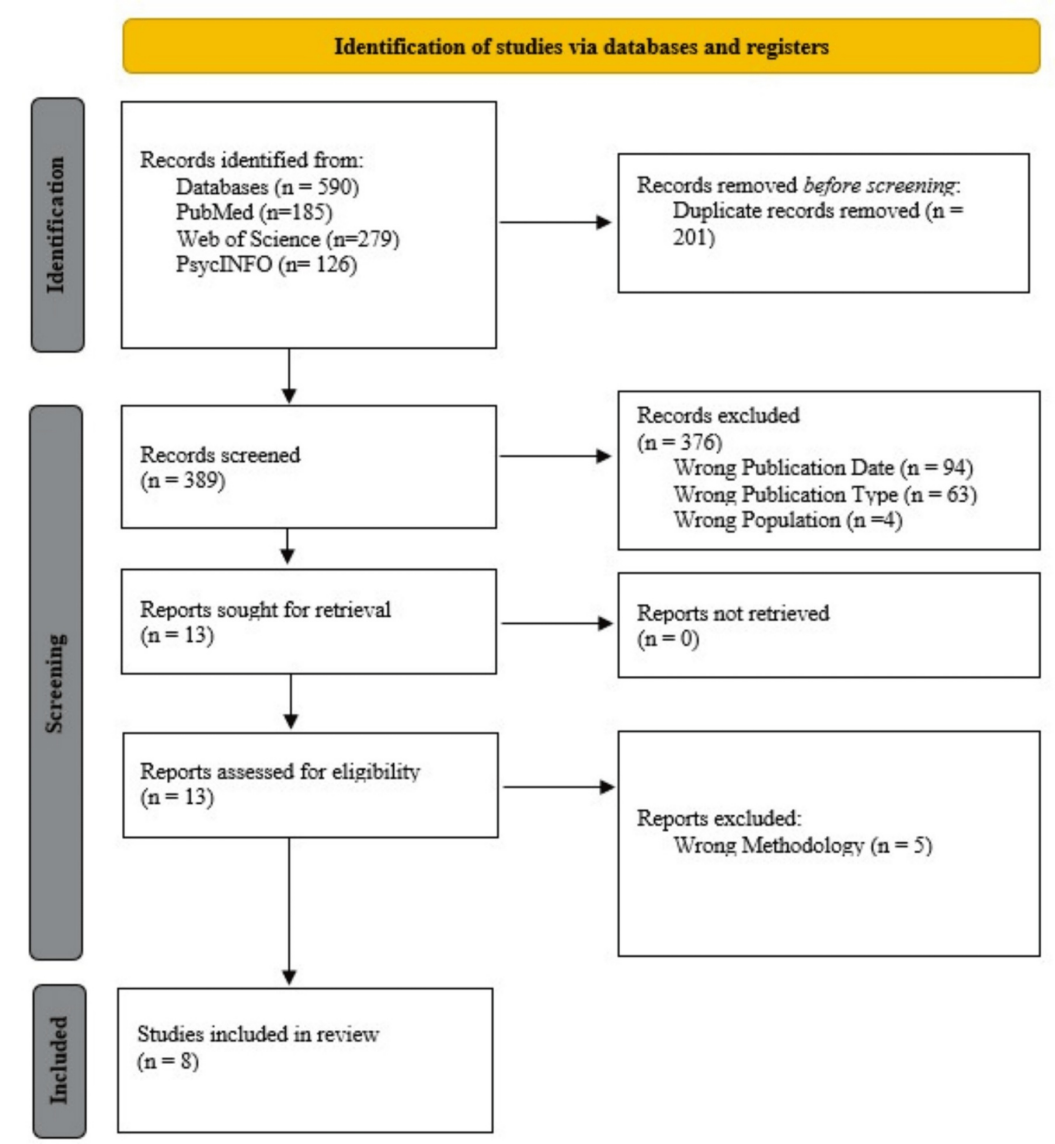
PRISMA diagram PRISMA: Preferred Reporting Items for Systematic Reviews and Meta-Analyses

This systematic review was conducted following the Preferred Reporting Items for Systematic Reviews and Meta-Analyses (PRISMA) guidelines (Figure 1). The study protocol was registered a priori with the International Prospective Register of Systematic Reviews (PROSPERO) (Record ID: CRD42025563610). PubMed, PsychINFO, and Web of Science were searched using a librarian-assisted search strategy (Table 1). Relevant search terms included "Schizophrenia", "Shared Decision Making", "Patient Outcome", "Patient Compliance", and "Quality of Life". Both MeSH terms and pertinent free-text keywords were used to ensure a broad and inclusive search. The search was limited to articles published from January 2015 onwards to capture significant advancements in mental health diagnostic criteria, treatment approaches, and the understanding of schizophrenia. Older studies were excluded to maintain relevance and comparability with current clinical practices.

**Table 1:** Search strategy

Database	Search Strategy	Results
PubMed	(("Schizophrenia"[MeSH] OR "Schizophrenia Spectrum and Other Psychotic Disorders"[MeSH] OR "Schizophrenia" OR "Schizophrenia"[tiab] OR "Schizophrenia Spectrum and Other Psychotic Disorders"[tiab]) AND ("Decision making, shared"[MeSH] OR "shared decision making" OR "shared decision making"[tiab]) AND ("Mental Health"[MeSH] OR "Treatment Adherence and Compliance"[MeSH] OR "Quality of Life"[MeSH] OR "Patient Compliance"[MeSH] OR "Symptom Assessment"[MeSH] OR "Hospitalization"[MeSH] OR "Patient Outcome Assessment"[MeSH] OR "Mental Health"[tiab] OR "Treatment Adherence and Compliance"[tiab] OR "Quality of Life"[tiab] OR "Patient Compliance"[tiab] OR "Symptom Assessment"[tiab] OR "Hospitalization"[tiab] OR "Patient Outcome Assessment"[tiab]))	185
Web of Science	((Schizophrenia OR "Schizophrenia Spectrum and Other Psychotic Disorders" OR Schizophrenia OR Schizophrenia OR "Schizophrenia Spectrum and Other Psychotic Disorders") AND ("Decision making, shared" OR "shared decision making") AND ("Mental Health" OR "Treatment Adherence and Compliance" OR "Quality of Life" OR "Patient Compliance" OR "Symptom Assessment" OR Hospitalization OR "Patient Outcome Assessment"))	279
PsychINFO	((exp Schizophrenia/ OR exp "Schizophrenia Spectrum and Other Psychotic Disorders"/ OR Schizophrenia OR Schizophrenia.ti,ab. OR "Schizophrenia Spectrum and Other Psychotic Disorders".ti,ab.) AND (exp "Decision making, shared"/ OR "shared decision making" OR "shared decision making".ti,ab.) AND (exp "Mental Health"/ OR exp "Treatment Adherence and Compliance"/ OR exp "Quality of Life"/ OR exp "Patient Compliance"/ OR exp "Symptom Assessment"/ OR exp Hospitalization/ OR exp "Patient Outcome Assessment"/ OR "Mental Health".ti,ab. OR "Treatment Adherence and Compliance".ti,ab. OR "Quality of Life".ti,ab. OR "Patient Compliance".ti,ab. OR "Symptom Assessment".ti,ab. OR Hospitalization.ti,ab. OR "Patient Outcome Assessment".ti,ab.))	126
Total		590

Duplicate records were removed, and two independent reviewers (AL and KJ) assessed the titles and abstracts using the Rayyan platform (Rayyan Systems Inc., Cambridge, MA). Discrepancies were resolved through discussion, with a third reviewer (SV) consulted when necessary. Full-text copies of relevant studies were retrieved for further eligibility assessment, which was again performed by two independent reviewers (AL and KJ), and any disagreements were resolved by consensus with the third reviewer (SV).

Selection Criteria

Studies were included if they 1) evaluated SDM interventions in patients with schizophrenia, with the requirement that the intervention was explicitly described or identified as SDM, irrespective of study design; 2) provided sufficient data on the primary outcome (i.e., treatment adherence) or secondary outcomes of interest (e.g., quality-of-life factors, such as symptom severity, self-esteem, autonomy); 3) included randomized controlled trials, quasi-experimental studies, or observational studies; 4) were published in English; and 5) were published in 2015 or later. Studies were excluded if they 1) were not published in English; 2) lacked details on SDM interventions or relevant outcome reporting; 3) were non-peer-reviewed literature; and 4) omitted both critical demographic (age, sex, race, ethnicity) and clinical (mental health status, symptom severity, hospitalization rate) information.

Data Extraction

Data were extracted independently by two reviewers (AL and KJ) and verified by a third reviewer (SV). Extracted information included study characteristics (e.g., title, authors, year, design, sample size, recruitment method), intervention details (e.g., SDM intervention type, duration), and primary or secondary outcome measures. For each outcome, numerical data were recorded as mean (± standard deviation) or as reported by the individual studies.

Outcome Measures

The primary outcome was treatment adherence, assessed by adherence rates or clinical outcomes. Secondary outcomes included mental health and quality-of-life factors: symptom severity, self-esteem, autonomy support, patient activation, problem-solving ability, self-efficacy, and patient-provider trust.

Data Analysis and Synthesis

A narrative synthesis was conducted to summarize the findings related to the impact of SDM on treatment adherence and secondary mental health outcomes due to high heterogeneity across methods and findings in the included papers. Studies evaluating SDM interventions in patients with schizophrenia assessed a range of outcomes, including self-esteem, quality of life, symptom severity, and problem-solving ability. Tools used included the World Health Organization Quality-of-Life Scele Abbreviated Version (WHOQOL-BREF), Positive and Negative Syndrome Scale (PANSS), Quality of Life Scale (QLS), 9-item Shared Decision Making Questionnaire (SDM-Q-9), Patient Satisfaction Index (PSI), Patient Activation Measure (PAM), and others to measure patient activation, medication adherence, therapeutic alliance, and satisfaction with care. Many studies specifically emphasized SDM and its impact on perceived involvement in care and preparedness for decision-making.

Quality Assessment

The quality of included studies was assessed using the modified Oxford Centre for Evidence-Based Medicine Rating. Appropriate quality assessment (QA) tools were used in accordance with the study design [13]. Risk of Bias in Non-Randomized Studies of Interventions (ROBINS-I) was utilized to assess bias for observational studies [14]. ROBINS-I evaluates bias across multiple domains, including confounding variables, selection biases, and outcome measurement [14].

## Review

Results

Key Sociodemographic Variables Across the Studies

Upon initial screening and removal of duplicates, 590 articles were screened, with 13 meeting the inclusion criteria (Figure 1). After meticulous analysis by three authors, eight articles were ultimately included involving a total of 1,861 participants [15-22]. Of these, 865 were in the intervention (SDM) group, and 996 were in the control group. The average age of participants in the SDM group was 34.73 years, compared to 31.00 years in the control group [16-22]. Notably, one study did not specify the mean age of participants [15].

Among the participants, 39.9% (n = 514) were female and 60.1% (n = 773) were male [15,16,18-22]. In the SDM group, 41.8% (n = 268) were female and 58.2% (n = 373) were male, while in the control group, 38.1% (n = 246) were female and 61.9% (n = 400) were male [15,16,18-22]. Notably, one study did not specify the sex of participants, which contributed to discrepancies in the total calculation [17]. Additionally, Browne et al. [16] and Robinson et al. [21] used overlapping datasets; thus, their participants were only counted once to avoid duplication (Table 2).

**Table 2:** Demographic data summary SDM: Shared decision-making

Key Demographics Across All Studies					
Variables	Average Age (Years)	Percent Female (%)	Percent Male (%)	Number of Females (n)	Number of Males (n)
SDM Group	34.73	41.8	58.2	268	373
Control Group	31.00	38.1	61.9	246	400
Total Study Participants	35.30	39.9	60.1	514	773

Regarding race and ethnicity, this information was reported in three studies [15,16,21]. Two of these studies collected data from the same RAISE-ETP trial; subsequently, their racial and ethnic data were also counted only once to avoid duplication [16,21]. Among participants in the SDM group, there were 29 Asian, 63 Black, 55 Hispanic, and 138 White individuals, with 22 categorized as “other” or “unspecified". The control group included 31 Asian, 89 Black, 18 Hispanic, and 80 White participants, with 12 categorized as “other” or “unspecified”. These findings provide insights into the racial and ethnic composition of participants included in SDM research; however, the limited number of studies reporting this data highlights gaps in demographic reporting (Table 3).

**Table 3:** Study overview DSM-IV-TR: Diagnostic and Statistical Manual of Mental Disorders, Fourth Edition, Text Revision; SDM: Shared decision-making

Authors	Year	Primary vs Substudy	Study Design	Country of Origin	Method of Data Analysis	Recruitment Method	Study Duration	Total Participants with Schizophrenia in the Study (n)	Total Participants in the SDM Group (n)	Schizophrenia Subtypes in the SDM Group (n/%)	Positive and Negative Schizophrenia Features of SDM Participants (%)	Total Participants in the Control Group (n/%)	Schizophrenia Subtypes in the Control Group (n/%)	Positive and Negative Schizophrenia Features of Control Group Participants (%)	Mean Age Across All Study Participants (years)	Mean Age in the SDM Group (years)	Mean Age in Control Group (years)	Total Females Across All Study Participants (n/%)	Total Females in the SDM Group (n/%)	Total Females in the Control Group (n/%)	Total Males Across All Study Participants (n/%)	Total Males in the SDM Group (n/%)	Total Males in the Control Group (n)	Total Non-binary Individuals (n/%)	Total Non-binary Individuals in the SDM Group (n/%)	Total Non-binary Individuals in the Control Group (n/%)	Race or Ethnicities in the SDM Group (n/%)	Race or Ethnicities in the Control Group (n/%)	Schizophrenia Diagnosis Measurement	SDM Intervention Description	SDM Intervention Types	Methods Used to Measure Schizophrenia Severity	Methods Used to Measure Secondary Outcomes	Methods Used to Measure SDM Treatment Adherence	Methods Used to Measure Medication Adherence	Primary and Secondary Outcomes Measured in the Study
An et al. [15]	2015	Original Study	Quasi-experiment with a nonequivalent control group pre-posttest design	South Korea	Chi-square test and independent t-tests	Hospital	April 2012 to May 2012	60	29	Schizophrenia: 27 (86.2%). Schizoaffective: 2 (6.9%)	NR	31	Schizophrenia: 30 (80.6%). Schizoaffective: 1 (3.2%)	NR	NR	≤29 years old (n=2) 6.9% 30–39 years old (n=5) 17.2% 40–49 years old (n=11) 37.9% ≥50 years old (n=11) 37.9%	≤29 years old (n=1) 6.9% 30–39 years old (n=3) 9.7% 40–49 years old (n=7) 22.6% ≥50 years old (n=20) 64.5%	0	0	0	60	29 (48.3%)	31 (51.7%)	NR	NR	NR	Asian: 29 (100%)	Asian: 31 (100%)	DSM-IV-TR criteria	Follow-up-based/therapy sessions consisted of eight group therapy sessions emphasizing effective communication and decision-making skills. Key components included role-playing to simulate patient-therapist interactions, the use of decision aids to inform participants about their options, and activities designed to enhance problem-solving abilities and communication skills.	Follow-up/Therapy SDM Sessions	NR	WHO Quality of Life Scale Abbreviated Version (WHOQOL-BREF), Rosenberg's Self-Esteem Scale, Problem-Solving Inventory	NR	NR	Primary: Self-esteem, problem-solving ability, and Quality of Life (QoL)
Browne et al. [16]	2017	Substudy from the RAISE ETP trial	Randomized cluster design	United States	Three-level conditioning linear growth modeling	Recruited from 17 NAVIGATE sites and 17 community care clinic sites	July 2010-July 2014	404	223	Schizophrenia: 101 (56%). Schizoaffective bipolar: 13 (7%). Schizoaffective depressive: 25 (14%). Schizophreniform: 24 (13%). Brief psychotic disorder: 1 (1%). Psychotic disorder: not otherwise specified 17 (9%)	Positive: 12.13%. Negative: 16.34%. Disorganized-concrete: 7.34%. Excited: 6.38%. Depressed: 7.93%	181	Schizophrenia: 113 (51%). Schizoaffective bipolar: 11 (5%). Schizoaffective depressive: 32 (14%). Schizophreniform: 43 (19%). Brief psychotic disorder: 1 (1%). Psychotic disorder not otherwise specified: 23 (10%)	Positive: 12.32%. Negative: 16.98%. Disorganized-concrete: 8.18%. Excited: 7.05%. Depressed: 8.16%	23.13	23.18	23.08	111 (27%)	61 (34%)	50 (22%)	293 (73%)	120 (66%)	173 (78%)	NR	NR	NR	Caucasian: 138 (62%). African American: 63 (28%). Other 22: (10%). Hispanic ethnicity: 55 (25%)	Caucasian: 80 (44%). African American: 89 (49%). Other: 12 (7%). Hispanic ethnicity: 18 (10%)	DSM-IV-TR criteria	NAVIGATE is a multielement treatment comprising indi- individualized medication management, family psychoeducation, resilience-focused individual therapy, and supported employment and education. In contrast to symptom-focused treatment, NAVIGATE is designed to promote recovery through a focus on the client as a person possessing strengths and resilience.	Medication Management, Family Psychoeducation, Resilience-focused Therapy, Employment and Education Support	The Positive and Negative Syndrome Scale (PANSS)	Quality of Life Scale (QLS)	Clinician-interviewers via two-way video conferencing	NR	Primary: Perceived autonomy support, QoL, symptom severity. Secondary: Relationship between autonomy support and outcomes.
Finnerty et al. [17]	2018	Substudy from MyCHOIS participants	Propensity score-matched intervention study	United States	Descriptive statistics, chi-square, t-tests, and multilevel linear models	Recruited from Medicaid-enrolled MyCHOIS–CommonGround with propensity score–matched adults receiving services in non-participating clinics	February 2011 - March 2014	564	189	NR	NR	375	NR	NR	43.9	43.8	44	Cannot be calculated (data not provided for schizophrenia subset; only total sample size available)	Cannot be calculated (data not provided for schizophrenia subset; only total sample size available)	Cannot be calculated (data not provided for schizophrenia subset; only total sample size available)	Cannot be calculated (data not provided for schizophrenia subset; only total sample size available)	Cannot be calculated (data not provided for schizophrenia subset; only total sample size available)	Cannot be calculated (data not provided for schizophrenia subset; only total sample size available)	NR	NR	NR	Cannot be calculated (data not provided for schizophrenia subset; only total sample size available)	Cannot be calculated (data not provided for schizophrenia subset; only total sample size available)	DSM-IV-TR criteria	CommonGround is one of the most widely implemented shared decision-making programs in mental health settings. This web-based tool guides clients through a questionnaire that results in a shared decision-making report summarizing the individual’s treatment concerns, goals, wellness strategies, and outcomes, which creates a foundation for shared decision-making discussions between the individual and his or her physician.	CommonGround SDM Model	NR	NR	MyCHOIS–CommonGround user logs	Medicational prescription fills	Primary: Engagement in mental health services. Secondary: Treatment adherence (for schizophrenia subgroup).
Harmann et al. [18]	2020	Substudy from the SDM-PLUS trial	Match-paired, cluster-randomized, non-blinded, controlled trial	Germany	Random-effects linear regression model	Recruited at the time of their admission to the psychiatric ward. All patients fulfilling the inclusion criteria were consecutively screened for the trial upon their admission​	October 2016 until March 2018​	322	161	Paranoid schizophrenia: 99 (61%). Schizoaffective: 52 (32%). Other schizophrenia diagnosis: 10 (6%)	NR	161	Paranoid schizophrenia: 115 (71%). Schizoaffective: 76 (47%). Other schizophrenia diagnosis: 8 (5%)	NR	41.75	42.1	41.4	160 (49.9%)	84 (52%)	76 (47%)	162 (50.1%)	77 (48%)	85 (53%)	NR	NR	NR	NR	NR	DSM-IV-TR criteria	Physicians were trained via workshops, supervision, and one-on-one teaching to ensure successful delivery of SDM. Patients were provided with group training in SDM and the use of question prompt sheets for ward rounds and individual consultations. This training occurred twice weekly and multiple workshops	Physician and Patient SDM Training with Question Prompt Sheets	Clinical Global Impression (CGI) and Global Assessment of Functioning (GAF), Insight Scale	Shared Decision making Q-9 (SDMQ9) Questionnaire; Questionnaire on Patients’ Treatment Satisfaction; Camberwell Assessment of Need Self-report Questionnaire (CANSAS-P); World Health Organization (WHO-5) well-being index and the EUROHIS-QOL scale	NR	MARS (Medication Adherence Rating Scale)	Primary: Perceived involvement in decision-making. Secondary: Therapeutic alliance, treatment satisfaction, treatment adherence.
Harmann et al. [19]	2016	Original Study	Randomized-controlled trial	Germany	Chi-squared test	Recruited in four psychiatric hospitals in Germany	October 2011 to April 2013	264	116	Schizophrenia: 76 (69%). Schizoaffective disorder: 31 (26%). Other: 3 (3%)	NR	99	Schizophrenia: 68 (72%). Schizoaffective disorder: 25 (26%). Other: 2 (2%)	NR	37.3	36.4	38.2	96	47 (41%)	49 (49%)	119	69 (59%)	50 (51%)	NR	NR	NR	NR	NR	DSM-IV-TR criteria	The SDM-training sessions included motivational (e.g., prospects of participation, patient rights) and behavioral aspects (e.g., role plays) and addressed important aspects of the patient–doctor interaction, such as question asking or giving feedback. The intervention was a 5-session training (60 min/session) addressing patient competencies for SDM	Motivational and Behavioral SDM Training	Clinical Global Impression (CGI) and Global Assessment of Functioning (GAF), Insight Scale	Treatment Satisfaction Scale, Autonomy Preference Index (API)	Physician Adherence Rating Scale, Patient Self-Reported Adherence Scale	Medication Adherence Questionnaire (MAQ)	Primary: Treatment adherence. Secondary: Attitudes towards decision-making, participation in consultations.
Perez-Revuelta et al. [20]	2023	Original Study	Single-blind, randomized controlled trial	Spain	Student’s t-test for independent samples and the chi-squared test	Recruited from a Mental Health Unit (MHU) of the public university hospital of the Andalusian Health Department	January 2014 to June 2017	102	51	Schizophrenia: 32 (64%). Schizoaffective: 18 (36%). First psychotic episode: 2 (4%)	NR	51	Schizophrenia: 37 (73%). Schizoaffective: 14 (27%). First psychotic episode: 5 (10%)	NR	42.24	40.22	44.23	27 (26.73%)	11 (22%)	16 (31.37%)	74 (73.27%)	39 (78%)	35 (68.63%)	NR	NR	NR	NR	NR	DSM-IV-R criteria	The experimental group participated in SDM sessions prior to discharge with regular booster sessions over the one-year follow-up. The health care team responsible for SDM was predisposed to concordance (LatCon II scale) and received specific training in SDM.	Pre- and Post-Discharge SDM Sessions with Booster Training	The Positive and Negative Syndrome Scale (PANSS) Insight Scale, Severity of Psychotic Symptoms Specific Scale	Drug Attitude Inventory (DAI), Working Therapeutic Alliance Inventory (WAI-S), The Combined Outcome Measure for Risk Communication and Treatment Decision Making Effectiveness (COMRADE)	Working Alliance Inventory Scale	Brief Adherence Rating Scale (BARS)	Primary: Treatment adherence, confidence, and satisfaction with decisions. Secondary: Symptom severity, days of hospitalization.
Robinson et al. [21]	2018	Substudy from the RAISE ETP trial	Match-paired, cluster-randomized, non-blinded, controlled trial	United States	Multiple comparisons adjustments	Recruited from 17 NAVIGATE sites and 17 community care clinic sites	July 2010-July 2014	404	223	Schizophrenia: 101 (56%). Schizoaffective bipolar: 13 (7%). Schizoaffective depressive: 25 (14%). Schizophreniform: 24 (13%). Brief psychotic disorder: 1 (1%). Psychotic disorder not otherwise specified: 17 (9%)	Positive: 12.13% Negative: 16.34%. Disorganized-concrete: 7.34%. Excited: 6.38%. Depressed: 7.93%	181	Schizophrenia: 113 (51%). Schizoaffective bipolar: 11 (5%). Schizoaffective depressive: 32 (14%). Schizophreniform: 43 (19%). Brief psychotic disorder: 1 (1%). Psychotic disorder not otherwise specified 23: (10%)	Positive: 12.32%. Negative: 16.98%. Disorganized-concrete: 8.18%. Excited: 7.05%. Depressed: 8.16%	23.13	23.18	23.08	111 (27%)	61 (34%)	50 (22%)	293 (73%)	120 (66%)	173 (78%)	NR	NR	NR	Caucasian: 138 (62%). African American: 63 (28%). Other: 22 (10%). Hispanic ethnicity: 55 (25%)	Caucasian: 80 (44%). African American: 89 (49%). Other: 12 (7%). Hispanic ethnicity: 18 (10%)	DSM-IV-TR criteria	NAVIGATE is a multielement treatment comprising individualized medication management, family psychoeducation, resilience-focused individual therapy, and supported employment and education. In contrast to symptom-focused treatment, NAVIGATE is designed to promote recovery through a focus on the client as a person possessing strengths and resilience.	Medication Management, Family Psychoeducation, Resilience-focused Therapy, Employment and Education Support	The Positive and Negative Syndrome Scale (PANSS)	Quality of Life Scale (QLS)	Clinician-interviewers via two-way video conferencing	NR	Primary: Treatment adherence, side effects, metabolic outcomes. Secondary: QOL, symptom severity).
Vitger et al. [22]	2022	Original Study	Randomized-controlled trial	Denmark	2-sample t test (2-tailed), chi-square test, and Fisher's exact test	Recruited from nine outpatient treatment sites	January 2019-March 2021	194	96	Schizophrenia: 68 (35.1%). Schizotypal: 102 (52.6%). Non-organic psychosis: 22 (11.3%). Schizoaffective: 2 (1%)	NR	98	Schizophrenia: 28 (29.2%). Schizotypal: 54 (56.3%). Non-organic psychosis: 14 (14.6%). Schizoaffective: 0 (0%)	NR	23.5	22.7	24.3	120 (61.9%)	65 (67.7%)	55 (56.1%)	65 (33.5%)	39 (39.8%)	26 (27.1%)	9 (4.6%)	5 (5.2%)	4 (4.1%)	NR	NR	DSM-IV-TR criteria	The digital SDM tool tested in this trial consisted of a smartphone app for the patient with functions such as preparation for consultation, daily self-assessments, action plans, and educational material.	Digital Smart Phone SDM	Clinical Interview, Scale for the Assessment of Positive Symptoms (SAPS), Scale for the Assessment of Negative Symptoms (SANS), Global Assessment of Functioning (GAF), and Personal Smartphone App, Social Performance Scale (PSP)	Adult State Hope Scale, Patient Activation Measure (PAM), General Self-Efficacy Scale (GSE), Perceived Efficacy in Patient-Physician Interactions (PEPPI) Questionnaire, Working Alliance Inventory–short form (WAI-S), Preparation for Decision-Making (PrepDM), and Client Satisfaction Questionnaire (CSQ).	NR	NR	Primary: Patient activation (self-reported). Secondary: Self-efficacy, confidence, satisfaction, symptom severity.

Study Design

Six studies were randomized controlled trials [16,18-22], one was a propensity score-matched intervention study [17], and one was a quasi-experimental study [15]. These studies evaluated a variety of SDM interventions across inpatient, outpatient, and digital settings delivered as scheduled medical appointments. These included recurring follow-up, therapy, routine medication management check-ins, family psychoeducation sessions, and employment or educational support meetings. Additional formats featured interventions that were timed around transitions of care, such as pre- and post-discharge SDM sessions.

Study Length

The duration of interventions and follow-up periods varied significantly among the studies, reflecting the diverse methodologies used to evaluate the impact of SDM. Short-term interventions, such as those in the Harmann et al.'s and Vitger et al.'s studies, assessed outcomes within four to eight weeks post intervention [19,22]. These shorter durations allowed for a focused examination of immediate changes in patient engagement, satisfaction, and communication improvements. For example, Vitger et al. evaluated digital SDM tools shortly after implementation, emphasizing their initial success in enhancing patient activation and provider-patient communication [22]. However, these studies were limited in their ability to determine whether such effects were sustained beyond the initial period.

In contrast, longer-term studies, such as Harmann et al. and Perez-Revuelta et al., adopted follow-up periods extending up to 12 months [18,20]. Their studies’ extended durations provided valuable insights into the long-term efficacy of SDM interventions, particularly regarding clinical outcomes such as symptom management and medication adherence. Perez-Revuelta et al., for example, highlighted that, while initial gains in patient engagement and satisfaction were evident, the benefits diminished over time without additional reinforcement or follow-up [20]. Similarly, Harmann et al. [18] evaluated outcomes at multiple intervals, revealing a gradual decline in adherence and quality of life measures over the 12-month period, bringing to light the importance of sustained intervention efforts.

The variation in follow-up periods across studies highlights the importance of tailoring SDM evaluation strategies to the desired outcomes: short-term assessments capture immediate impacts, while longer ones assess sustainability and broader outcome. Designing SDM programs with built-in mechanisms for reinforcement and periodic reassessment may be crucial to maintaining their benefits over time.

Measurement Tools and Validation Scales

Despite differences in duration, all studies employed validated tools. For example, An et al. utilized the Rosenberg Self-Esteem Scale (RSE) to assess self-esteem, the WHOQOL-BREF to evaluate quality of life, and the Problem-Solving Inventory (PSI) to measure problem-solving ability [15]. Browne et al. employed the PANSS and the QLS to evaluate active symptoms and quality of life [16]. Similarly, Robinson et al. used the PANSS and the QLS while conducting clinical interviews to track medication adherence [21]. Table 4 presents a summary of this information across all studies. Detailed observations are provided in the results section.

**Table 4:** Narrative analysis summary QOL: Quality of life

Summary of Key Findings Across All Studies		
Author	Outcomes Measured	Key Findings
An et al. [15]	Primary: Self-esteem, problem-solving ability, and QoL	The SDM training significantly improved self-esteem, problem-solving ability, and QoL.
Browne et al. [16]	Primary: Perceived autonomy support, QoL, symptom severity. Secondary: Relationship between autonomy support and outcomes	NAVIGATE participants showed greater perceived autonomy support, which was associated with higher QoL and improved symptoms across both treatment groups.
Finnerty et al. [17]	Primary: Engagement in mental health services. Secondary: Treatment adherence (for schizophrenia subgroup)	SDM tool users had higher engagement in outpatient services (8.5 vs. 6.9 months of service) and higher antipsychotic adherence.
Harmann et al. [18]	Primary: Perceived involvement in decision-making. Secondary: Therapeutic alliance, treatment satisfaction, treatment adherence	SDM-PLUS had no impact on adherence or rehospitalization, but it significantly improved patient involvement, therapeutic alliance, and treatment satisfaction.
Harmann et al. [19]	Primary: Treatment adherence. Secondary: Attitudes towards decision-making, participation in consultations	No long-term effect on adherence but significant short-term improvements in patient participation in psychiatric consultations and decision-making preferences.
Perez- Revuelta et al. [20]	Primary: Treatment adherence, confidence, and satisfaction with decisions. Secondary: Symptom severity, days of hospitalization	SDM with booster sessions significantly improved adherence and satisfaction with treatment. Regression analysis indicated that more booster sessions were associated with better adherence.
Robinson et al. [21]	Primary: Treatment adherence, side effects, metabolic outcomes. Secondary: QOL, symptom severity)	NAVIGATE participants experienced better adherence, fewer side effects, and improved QoL. They were more likely to receive antipsychotics conforming to guidelines, had fewer beliefs related to non-adherence, and gained less weight.
Vitger et al. [22]	Primary: Patient activation (self-reported). Secondary: Self-efficacy, confidence, satisfaction, symptom severity	The digital SDM tool significantly improved patient activation, confidence in communication with providers, and decision-making preparedness. No significant effect on treatment satisfaction, symptom severity, or functioning.

Harmann et al. assessed treatment adherence, attitudes toward decision-making, and satisfaction with consultations using scales such as the Clinical Global Impressions Scale (CGI), Global Assessment of Functioning (GAF), Insight Scale, Treatment Satisfaction Scale, and the Medication Adherence Questionnaire (MAQ) [18]. They focused on perceived involvement in decision-making, therapeutic alliance, and quality of life, employing the SDM Questionnaire-9 (SDMQ9), Camberwell Assessment of Need Short Appraisal Scale-Patient Version (CANSAS-P), and the EUROHIS-QOL scale, amongst others [19].

Vitger et al. utilized several scales to assess the outcomes of their digital SDM tool, including the PAM to evaluate patient activation, Perceived Efficacy in Patient-Physician Interactions (PEPPI) to measure confidence in communication with providers, and the Prepared Decision Making (PrepDM) to assess decision-making preparedness [22]. Perez-Revuelta et al. implemented the Brief Adherence Rating Scale (BARS), PANSS, and Working Alliance Inventory-Short (WAI-S) to assess treatment adherence, symptom severity, and therapeutic alliance [20]. Finally, Finnerty et al. relied on the MyCHOIS platform to track medication adherence and engagement in mental health services [17].

SDM Interventions

SDM tools were employed through various modalities across the studies. For example, digital models were integrated into two studies, highlighting a trend toward incorporating technologies in SDM interventions [17,22]. Finnerty et al. employed a web-based decision aid to support patient participation in treatment decisions, while Vitger et al. used a smartphone application with features like symptom tracking, medication reminders, and interactive decision-making support [17,22]. These tools were designed to enhance patient activation and communication, enabling engagement through technological interventions.

In contrast, Browne et al. and Robinson et al. implemented the SDM model NAVIGATE, which included structured psychoeducation, collaborative goal setting, and follow-up support to promote autonomy and improve outcomes related to symptom management and medication adherence [16,21]. Similarly, Perez-Revuelta et al. utilized a tailored SDM program with the goal of enhancing medication adherence and symptom management in patients with schizophrenia [20].

For training-based interventions, An et al. implemented SDM patient training to improve patient engagement, self-esteem, and quality of life by equipping healthcare providers with techniques to foster better communication and collaboration with patients [15]. Finnerty et al. integrated SDM training into a broader educational program on schizophrenia and treatment options, empowering patients to make informed decisions about their care [17]. Similarly, Harmann et al.’s studies conducted SDM training for both patients and clinicians, aiming to improve treatment adherence and self-management [18,19]. Additionally, Harmann et al.’s research explored hybrid models that combined SDM training with traditional therapy approaches to evaluate both short- and long-term treatment adherence [18,19].

These studies highlight the adaptability and broader implications of SDM interventions, which range from digital tools to personalized programs and focused training efforts, demonstrating that diverse approaches can enhance patient engagement and improve clinical outcomes in schizophrenia across various care settings.

Study Outcomes

Results of this systematic review demonstrated significant short-term benefits of SDM interventions in improving patient engagement [16,20], treatment adherence [15-19], quality of life [13,14,16,19], and satisfaction [16,20], particularly among populations with schizophrenia and related conditions. Specifically, An et al. reported substantial increases in self-esteem (+4.06) and quality of life (+8.03 points on the WHOQOL-BREF) among patients who received SDM training compared to controls [15]. Additionally, structured SDM programs, such as NAVIGATE, demonstrated positive outcomes. Results from et al.'s and Robinson et al.'s studies found that these programs not only enhanced autonomy and patient engagement but also led to tangible improvements in clinical management, including symptom control, medication adherence, and reduced side effects in psychosis patients [16,21]. Perez-Revuelta also reported improvements in decision-making and greater patient satisfaction, although long-term adherence remained mixed [20]. The success of these programs appears to have stemmed from their comprehensive design, integrating psychoeducation, collaborative goal setting, and follow-up support.

The role of technologies in SDM has also garnered attention. For example, Finnerty et al. illustrated that web-based SDM training enhanced patient involvement in treatment decisions, resulting in better medication adherence and symptom reduction [17]. Similarly, Vitger et al.’s study, which employed a smartphone-based SDM intervention, demonstrated significant improvements in patient activation, communication confidence, and decision-making preparedness [22]. Despite these advancements, Vitger et al. noted minimal improvements in clinical outcomes such as symptom severity, daily functioning, and hospitalization rates [22]. This discrepancy suggests that, while digital-only interventions can effectively enhance engagement, they may need to be integrated with traditional care models to influence broader clinical metrics.

Moreover, the variability in outcomes across studies suggests that SDM efficacy may depend on the intensity and duration of interventions. For instance, longer-term studies, such as those by Harmann et al. and Perez-Revuelta et al., found no significant improvements in medication adherence after 12 months without sustained reinforcement [18-20]. Thus, it can be reasoned that the benefits of SDM may diminish over time if not actively maintained (Tables 5-6).

**Table 5:** Study characteristics Note: The data from Browne et al. and Robinson et al. were substudies extracted from the randomized controlled trial RAISE ETP SDM: Shared decision-making; QOL: Quality of life

Key Study Characteristics and Findings Across All Studies														
Author	Date Published	Study's Country of Origin	Study Design	Study Duration	Total Participants in SDM group (n)	Total Participants in Control group (n)	Primary Study vs Substudy	Methods Used to Measure Primary Outcomes (Medication Adherence)	Methods Used to Measure Secondary Outcomes (QOL, Mental Health)	Methods Used to Measure Schizophrenia Symptom Severity	SDM Intervention Types	Methods Used to Measure SDM Treatment Adherence	Primary & Secondary Outcomes	Study's Key Findings
An et al. [15]	2015	South Korea	Quasi-experiment with a nonequivalent control group pre-posttest design	April 3, 2012 to May 29, 2012	29	31	Original Study	NR	WHO Quality of Life Scale Abbreviated Version, Rosenberg's Self-Esteem Scale, Problem-Solving Inventory	NR	Follow-up/Therapy SDM Sessions	NR	Primary: Self-esteem, problem-solving ability, and QoL	The SDM training significantly improved self-esteem, problem-solving ability, and QoL.
Browne et al. [16]	2017	United States	Randomized cluster design	July 2010-July 2014	223	181	Substudy from the RAISE ETP trial	NR	Quality of Life Scale	The Positive and Negative Syndrome Scale	Medication Management, Family Psychoeducation, Resilience-focused Therapy, Employment and Education Support	Clinician-interviewers via two-way video conferencing	Primary: Perceived autonomy support, QoL, symptom severity. Secondary: Relationship between autonomy support and outcomes.	NAVIGATE participants showed greater perceived autonomy support, which was associated with higher QoL and improved symptoms across both treatment groups.
Finnerty et al. [17]	2018	United States	Propensity score-matched intervention study	February 1, 2011, to March 1, 2014	189	375	Substudy from MyCHOIS participants	Medicational prescription fills	NR	NR	CommonGround SDM Model	MyCHOIS–CommonGround user logs	Primary: Engagement in mental health services. Secondary: Treatment adherence (for schizophrenia subgroup).	SDM tool users had higher engagement in outpatient services (8.5 vs. 6.9 months of service). Among those with schizophrenia, SDM users had higher antipsychotic adherence (0.78 vs. 0.69, p < 0.01).
Harmann et al. [18]	2020	Germany	Match-paired, cluster-randomized, non-blinded, controlled trial	October 2016 until March 2018​	161	161	Substudy from the SDM-PLUS trial	Medication Adherence Rating Scale	Shared Decision Making Q-9 Questionnaire; Questionnaire on Patients’ Treatment Satisfaction; Camberwell Assessment of Need Self-report Questionnaire, World Health Organization-5 well-being index, and the EUROHIS-QOL scale	Clinical Global Impression Scale, Global Assessment of Functioning Scale, Insight Scale	Physician and Patient SDM Training with Question Prompt Sheets	NR	Primary: Perceived involvement in decision-making. Secondary: Therapeutic alliance, treatment satisfaction, treatment adherence.	SDM-PLUS significantly increased patient involvement in decision-making and improved therapeutic alliance and treatment satisfaction during inpatient stay. No significant impact on adherence or rehospitalization at 6- and 12-month follow-up.
Harmann et al. [19]	2016	Germany	Randomized-controlled trial	October 2011 to April 2013	116	99	Original Study	Medication Adherence Questionnaire	Treatment Satisfaction scale, Autonomy Preference Index (API)	Clinical Global Impression Scale, Global Assessment of Functioning Scale, Insight Scale	Motivational and Behavioral SDM Training	Physician Adherence Rating Scale, Patient Self-Reported Adherence Scale	Primary: Treatment adherence. Secondary: Attitudes towards decision-making, participation in consultations.	No long-term effect on adherence but significant short-term improvements in patient participation in psychiatric consultations and decision-making preferences.
Perez-Revuelta et al. [20]	2023	Spain	Single-blind, randomized controlled trial	January 2014 to June 2017	51	51	Original Study	Brief Adherence Rating Scale	Drug Attitude Inventory, Working Therapeutic Alliance Inventor, The Combined Outcome Measure for Risk Communication and Treatment Decision Making Effectiveness	The Positive and Negative Syndrome Scale, Insight Scale, Severity of Psychotic Symptoms Specific Scale	Pre- and Post-Discharge SDM Sessions with Booster Training	Working Alliance Inventory Scale	Primary: Treatment adherence, confidence, and satisfaction with decisions. Secondary: Symptom severity, days of hospitalization.	SDM with booster sessions significantly improved adherence and satisfaction with treatment. Regression analysis indicated that more booster sessions were associated with better adherence.
Robinson et al. [21]	2018	United States	Match-paired, cluster-randomized, non-blinded, controlled trial	July 2010-July 2014	223	181	Substudy from the RAISE ETP trial	NR	Quality-of-Life Scale	The Positive and Negative Syndrome Scale	Medication Management, Family Psychoeducation, Resilience-focused Therapy, Employment and Education Support	Clinician-interviewers via two-way video conferencing	Primary: Treatment adherence, side effects, metabolic outcomes. Secondary: QOL, symptom severity).	NAVIGATE participants experienced better adherence, fewer side effects, and improved QoL. They were more likely to receive antipsychotics conforming to guidelines, had fewer beliefs related to non-adherence, and gained less weight.
Vitger et al. [22]	2022	Denmark	Randomized-controlled trial	January 2019-March 2021	96	98	Original Study	NR	Adult State Hope Scale, General Self-Efficacy Scale, Perceived Efficacy in Patient-Physician Interactions Questionnaire, Working Alliance Inventory–short form, Preparation for Decision-Making, and Client Satisfaction Questionnaire.	Clinical Interview, Global Assessment of Functioning Scale, Scale for the Assessment of Positive Symptoms, Scale for the Assessment of Negative Symptoms	Digital Smart Phone SDM	NR	Primary: Patient activation (self-reported). Secondary: Self-efficacy, confidence, satisfaction, symptom severity.	The digital SDM tool significantly improved patient activation, confidence in communication with providers, and decision-making preparedness. No significant effect on treatment satisfaction, symptom severity, or functioning.

**Table 6:** Statistical outcomes SDM: Shared decision-making; PANSS: Positive and Negative Syndrome Scale

Author	Date Published	Primary & Secondary Outcomes Measured	Primary and Secondary Outcomes	Study's Conclusion
An et al. [15]	2015	Primary: Self-esteem, problem-solving ability, and Quality of Life (QoL)	The change in the quality of life measured using the WHO Quality of Life Scale Abbreviated Version (WHOQOL-BREF), scores of the intervention group (72.72 before to 80.75 after, an increase of 8.03) was significantly greater than that of the control group (72.29 before to 72.32 after, an increase of .03) (t = −3.40, p = .002). The change in self-esteem scores of the intervention group (25.31 before intervention to 29.37 after, producing an increase of 4.06) was significantly greater than that of the control group (26.12 before to 25.06 after, a decrease of −1.06) (t = −4.95, p < .001). The SDM training program significantly improved self-esteem, problem-solving ability, and quality of life in patients with schizophrenia. This suggests SDM can be effective in enhancing self-regard and subjective well-being.	SDM training is an effective tool for enhancing self-regard and QoL in schizophrenia, and it can be adapted for use in various mental health settings.
Browne et al. [16]	2017	Primary: Perceived autonomy support, QoL, symptom severity. Secondary: Relationship between autonomy support and outcomes	Higher autonomy support, facilitated by SDM, was significantly associated with improved quality of life and better symptom management in NAVIGATE participants compared to community care. Over time, autonomy support increased in NAVIGATE (slope = 0.08, t = 3.17, p = .002) but showed no significant change in community care (slope = –0.022). Improved autonomy support correlated with higher quality of life at baseline, 6, 12, and 18 months for NAVIGATE participants and at 12, 18, and 24 months in community care, highlighting stronger early benefits in NAVIGATE and delayed effects in community care. For symptoms, higher autonomy support was associated with lower overall PANSS scores (t = 22.82, df = 825, p = .005) and reduced excitement-related symptoms (t = 22.28, df = 920, p = .023) across both groups. A significant interaction (t = 22.31, df = 1,287, p = .021) revealed autonomy support's impact on depression-related symptoms, with lower depression scores at baseline in community care (t = 22.01, df = 1,287, p = .045). However, this effect was not sustained at later time points, nor was it observed in NAVIGATE. Additionally, autonomy support did not significantly affect positive, negative, or disorganized symptom scores in either group. In summary, perceived autonomy support improved quality of life and some symptoms, with the strongest early effects in NAVIGATE and delayed benefits in community care. However, its impact on depression-related symptoms was limited and not consistent over time.	Autonomy support, facilitated by SDM, improves QoL and symptom management, particularly in early intervention programs.
Finnerty et al. [17]	2018	Primary: Engagement in mental health services. Secondary: Treatment adherence (for schizophrenia subgroup)	Use of the web-based SDM tool improved engagement in mental health services and antipsychotic adherence in schizophrenia patients. The tool helped facilitate patient participation in treatment decisions. There were no differences in antipsychotic medication adherence between the groups at baseline, but the MyCHOIS–CommonGround group was significantly more adherent during the follow-up year compared with the control group (PDC, .78 versus .69; p=.01).	Web-based SDM tools can enhance treatment engagement and improve medication adherence in schizophrenia.
Harmann et al. [18]	2020	Primary: Perceived involvement in decision-making. Secondary: Therapeutic alliance, treatment satisfaction, treatment adherence	SDM-PLUS, an intervention tailored for acute schizophrenia patients, significantly increased perceived involvement in decision-making during inpatient stays (mean group difference = 16.5, 95% CI 9.0–24.0, p = 0.002; adjusted β = 17.3, 95% CI 10.8–23.6, p = 0.0004). Participants in the intervention group also reported improved therapeutic alliance, higher treatment satisfaction, and better self-rated medication compliance compared to the control group. However, no significant improvements were observed in long-term outcomes such as adherence, quality of life, or rehospitalization rates during 6- and 12-month follow-ups. While SDM-PLUS demonstrated positive effects on patient engagement and satisfaction during the inpatient period, these benefits did not translate into sustained improvements post-discharge.	SDM can improve patient involvement and satisfaction but may not affect long-term adherence in acute inpatient settings.
Harmann et al. [19]	2016	Primary: Treatment adherence. Secondary: Attitudes towards decision-making, participation in consultations	Short-term improvements in patient participation in psychiatric consultations were observed after SDM training. However, there were no long-term effects on treatment adherence at 6- and 12-month follow-up. The number of patients judged as having ‘‘good adherence’’ at 12 months after discharge was not significantly different between the intervention group (n = 51 of 91, 56%) and the control group (n = 49 of 82, 60%, v2 = 0.24, p = 0.62). When controlling for duration of illness, there was still no significant group difference regarding treatment adherence.	SDM training increases patient participation in consultations but does not lead to long-term improvements in adherence.
Perez-Revuelta et al. [20]	2023	Primary: Treatment adherence, confidence, and satisfaction with decisions. Secondary: Symptom severity, days of hospitalization	The study found no significant difference in treatment adherence between the control and experimental groups when assessed without numerical measures. However, a significant regression model (adjusted R² = 0.384; F[df=6] = 4.386; p < 0.001) identified a direct and independent association between adherence and the number of booster sessions in the SDM group, emphasizing the importance of reinforcing SDM to sustain adherence over time. Psychotic symptom severity (DSM-5 scale) decreased significantly from baseline to 12 months in both groups, with a moderate effect size, although no between-group differences in PANSS scores were observed after adjusting for baseline differences. The experimental group showed a greater reduction in DSM-5 scores at 12 months compared to discharge, a pattern not observed in the control group. Furthermore, no losses to follow-up were recorded in the experimental group, in contrast to a 19.6% loss in the control group (RR = 0.80, 95% CI = 0.70–0.92), highlighting the potential of SDM with booster sessions to enhance participant retention and engagement.	SDM with sustained reinforcement can improve long-term adherence and patient satisfaction in schizophrenia.
Robinson et al. [21]	2018	Primary: Treatment adherence, side effects, metabolic outcomes. Secondary: QOL, symptom severity)	The NAVIGATE program, which incorporates SDM, demonstrated better treatment adherence, reduced side effects, and less weight gain compared to standard care. It also improved quality of life while reducing psychosis and depressive symptoms. Over the two-year trial, NAVIGATE participants had significantly more medication visits (treatment-by-time interaction F = 3.78, p < 0.0001; effect of treatment F = 12.80, p = 0.0003), with an estimated monthly average of 0.554 visits (95% CI: 0.423, 0.685) compared to 0.292 visits (95% CI: 0.226, 0.357) in the Community Care group. NAVIGATE participants were also more likely to receive prescriptions aligned with program first-line principles (odds ratio = 2.189, 95% CI: 1.084, 4.421). Among those whose initial prescriptions did not conform to NAVIGATE principles, 62.7% in the NAVIGATE group eventually transitioned to first-line prescriptions compared to 44.4% in the Community Care group (odds ratio = 2.065, 95% CI: 1.024, 4.164, p = 0.0432). These findings highlight the effectiveness of the NAVIGATE program in optimizing treatment adherence and aligning prescriptions with evidence-based guidelines.	Comprehensive SDM-based interventions can enhance adherence, reduce side effects, and improve QoL in first-episode psychosis.
Vitger et al. [22]	2022	Primary: Patient activation (self-reported). Secondary: Self-efficacy, confidence, satisfaction, symptom severity	A digital tool supporting SDM significantly improved patient activation (mean difference = 4.39, 95% CI: 0.99–7.79, Cohen d = 0.33, p = .01), confidence in communication with providers (mean difference = 1.85, 95% CI: 0.01–3.69, Cohen d = 0.24, p = .05), and preparedness for decision-making (mean difference = 5.12, 95% CI: 0.16–10.08, Cohen d = 0.27, p = .04). These results demonstrate the tool’s efficacy in fostering patient engagement and collaborative care. However, no significant effects were observed on treatment satisfaction, hope, self-efficacy, working alliance, symptom severity, level of functioning, antipsychotic medication use, or psychiatric hospitalizations. While the tool enhances specific aspects of patient-provider interactions and decision-making, its broader impact on clinical and functional outcomes remains limited.	Digital tools can improve patient activation and communication but may not influence clinical outcomes like symptom severity or functioning.

Discussion

SDM interventions were associated with short-term improvements in treatment adherence, patient engagement, and perceived autonomy [17,20-22]. Additionally, increased self-esteem and quality of life observed in several studies suggest that these psychosocial improvements may play a role in fostering continued adherence [15,16,21].

Nonadherence in schizophrenia is shaped by multiple factors, including complex drug regimens, provider distrust, impaired executive judgment, and environmental barriers, such as stigma and financial constraints [23-26]. Moreover, patients often express feeling excluded from treatment decisions [27]. Traditional pharmacological interventions alone may not fully address these challenges [23-26]. SDM provides a holistic approach by promoting patient autonomy and collaborative treatment planning, which may be particularly effective in bridging these gaps [27].

Structured SDM programs have demonstrated improvements in both adherence and clinical outcomes, with evidence suggesting that engagement strategies tailored to different phases of treatment - including acute inpatient care - can be especially beneficial [19]. In acute settings, where patients often face heightened distress and limited decision-making capacity, SDM interventions can provide a sense of agency, strengthen the therapeutic alliance, and establish a foundation for sustained adherence after discharge [19,28].

Optimizing SDM for Sustained Adherence

While SDM interventions improve short-term engagement, their long-term efficacy remains mixed. Digital SDM tools, such as those evaluated by Harmann et al. and Vitger et al., improved patient activation but had limited impact on sustained adherence, aligning with prior research [12,19,22,29,30]. Given the observed decline in adherence without reinforcement, future SDM models should integrate sustained engagement strategies, such as booster sessions or structured psychoeducation and follow-up support [16,18,20,21]. These strategies may strengthen adherence by reinforcing SDM as an ongoing process rather than a single intervention.

Given the rapid development of digital health technologies, the role of digital SDM tools in mental health remains an emerging area of research. Conflicting findings highlight the complexity of their impact: while Stein et al. found that CommonGround, a widely implemented web-based SDM program, did not significantly improve medication adherence, Finnerty et al. demonstrated that other digital SDM interventions enhanced both medication adherence and engagement in outpatient mental health services [17,31]. These discrepancies suggest that the effectiveness of digital SDM tools may depend on specific design features, patient populations, or implementation strategies. Further research is needed to identify which digital components most effectively support SDM and how to integrate these tools seamlessly into traditional care models [19,22].

Considering the challenges of medication nonadherence in schizophrenia, our findings contribute to the growing body of literature on patient-centered psychiatric care. By synthesizing evidence across multiple SDM interventions, this review highlights how structured psychoeducation, digital tools, and sustained engagement strategies can enhance both short- and long-term adherence. Identifying key facilitators and barriers to SDM implementation provides actionable insights for improving clinical workflows, tailoring patient interventions, and shaping policy frameworks. These efforts are crucial to ensuring that SDM becomes a cornerstone of routine schizophrenia care, ultimately improving patient outcomes and quality of life.

The included studies in this review revealed notable variability in the design, scope, and outcomes of SDM interventions for individuals with schizophrenia. Most interventions were short-term and implemented in outpatient or community-based settings, with few addressing acute or inpatient care, where treatment engagement is often critical. While many studies demonstrated improvements in patient satisfaction, communication, and short-term adherence, there was a general lack of long-term follow-up to evaluate sustained clinical impact. Additionally, the diversity of patient populations was limited; individuals with severe symptoms or cognitive impairments were often underrepresented, despite their potential to benefit significantly from SDM approaches. Measurement tools and outcome reporting also varied widely, limiting comparability across studies. Based on these findings, future research should aim to standardize outcome measures, utilize longitudinal designs to evaluate sustainability, and include more diverse and underserved populations. Research should also examine SDM’s role in acute care settings and explore the most effective components of digital tools.

These findings have meaningful implications for healthcare providers, organizations, and policymakers who seek to promote SDM in mental healthcare. Providers should receive training in culturally competent communication and supported decision-making techniques, especially when working with patients with severe mental illness. Healthcare organizations may need to adapt workflows to accommodate SDM interventions and invest in digital decision aids that can be integrated into electronic health records. Policymakers can support SDM by incentivizing its adoption through reimbursement models and funding research that evaluates SDM effectiveness in diverse care settings. Overall, promoting SDM can improve treatment adherence, foster patient autonomy, and ultimately lead to better clinical outcomes in schizophrenia care. However, a system-wide commitment is essential to ensure SDM is accessible, equitable, and sustainable

Limitations/Future Directions

Our review has several limitations. First, publication bias was introduced by limiting the inclusion criteria to full-text studies published in English, potentially excluding relevant findings from gray literature or non-English sources. Additionally, recruitment biases were observed, as some studies selected participants with greater capacity for SDM, limiting generalizability to broader psychiatric populations. Another key limitation was the heterogeneity in data reporting, particularly regarding differences in provider-patient interactions, variations in SDM implementation, and inconsistencies in follow-up duration. These factors may have influenced study outcomes and made it difficult to draw uniform conclusions.

Future research should prioritize randomized controlled trials with standardized outcome measures to better assess SDM’s effectiveness in schizophrenia care. Furthermore, greater demographic diversity in SDM research is needed to explore whether certain populations experience differential benefits from these interventions. Understanding these variations could lead to more tailored, culturally responsive SDM models that improve accessibility and impact across diverse patient populations [32].

Additionally, findings highlight the need for structured, ongoing support and long-term follow-up rather than one-time interventions. Incorporating SDM into routine psychiatric care - whether through regular follow-ups, patient education, or digital engagement tools - could help bridge gaps in adherence and improve treatment success. Given the chronic nature of schizophrenia, sustained SDM efforts may empower patients to take on a more active role in their care, enhance functional status, and support cognitive and psychosocial recovery. By improving treatment adherence and self-management, SDM may also reduce caregiver burden, leading to better long-term patient and community outcomes.

## Conclusions

Our review suggests that SDM interventions are associated with enhanced treatment adherence in individuals with schizophrenia. Additionally, SDM interventions may correlate with improved secondary mental health factors, such as quality of life, self-esteem, and patient-provider trust. However, outcomes vary based on intervention style, duration, and intensity. While short-term SDM interventions showed positive effects, long-term and digital SDM interventions produced mixed or incomplete results. These findings illustrate the importance of further research to investigate how various SDM approaches, particularly those with ongoing support and patient engagement strategies, can be effectively integrated into psychiatric care.

By fostering collaboration and patient autonomy, SDM interventions have the potential to improve adherence, enhance patient-provider relationships, and ultimately contribute to a higher quality of life for individuals living with schizophrenia.
